# Vortioxetine Modulates the Regional Signal in First-Episode Drug-Free Major Depressive Disorder at Rest

**DOI:** 10.3389/fpsyt.2022.950885

**Published:** 2022-06-29

**Authors:** Shihong Xiong, Wei Li, Yang Zhou, Hongwei Ren, Guorong Lin, Sheng Zhang, Xi Xiang

**Affiliations:** ^1^Department of Nephrology, Tianyou Hospital Affiliated to Wuhan University of Science and Technology, Wuhan, China; ^2^Department of Otolaryngology-Head and Neck Surgery, Wuhan Asia General Hospital, Wuhan, China; ^3^Wuhan Mental Health Center, Wuhan, China; ^4^Department of Medical Imaging, Tianyou Hospital Affiliated to Wuhan University of Science and Technology, Wuhan, China; ^5^Liyuan Hospital of Tongji Medical College, Huazhong University of Science and Technology, Wuhan, China; ^6^Department of Spine and Orthopedics, Tianyou Hospital Affiliated to Wuhan University of Science and Technology, Wuhan, China

**Keywords:** major depressive disorder, MRI, amplitude of low-frequency fluctuation, vortioxetine, temporal lobe

## Abstract

**Background:**

Previous studies on brain functional alterations associated with antidepressants for major depressive disorder (MDD) have produced conflicting results because they involved short treatment periods and a variety of compounds.

**Methods:**

Resting-state functional magnetic resonance imaging scans were obtained from 25 first-episode drug-free patients with MDD and 25 healthy controls. The patients, who were treated with vortioxetine for 8 weeks, were scanned at two-time points (baseline and week 8 of treatment). The amplitude of low-frequency fluctuation (ALFF) in the imaging data was used to analyze local brain signal alterations associated with antidepressant treatment.

**Results:**

Compared with the controls, the patients at baseline showed decreased ALFF values in the right inferior temporal gyrus and increased ALFF values in the left inferior cerebellum, right cingulate gyrus and postcentral gyrus. After 8 weeks of vortioxetine treatment, patients showed increased ALFF values in the bilateral cingulate gyrus, middle temporal gyrus, medial superior frontal gyrus, and inferior cerebellum.

**Conclusion:**

This study provided evidence that vortioxetine modulates brain signals in MDD sufferers. These findings contribute to the understanding of how antidepressants effect brain function.

## Introduction

Major depressive disorder (MDD) is a global psychiatric disorder characterized by low mood, decreased interest, and loss of pleasure accompanied by marked cognitive decline (1–4). According to statistics from the World Health Organization, as of 2015, there were about 322 million people afflicted with depression in the world, accounting for 4.4% of the total population. There are more than 54 million MDD patients in China, and the lifetime prevalence rate is 6.9%. MDD was the third leading cause of disability globally in 2017 and is projected to be the predominant disease burden by 2030 (5). MDD leads to obvious impairments in cognitive functions, such as executive function, memory, and learning (6, 7). Cognitive functions refer to processes that occur when acquiring, encoding, manipulating, extracting, and using sensory input during the cognition of objective things and includes perception, memory, thinking, and attention (8). Impaired cognitive function seriously affects the MDD patients’ ability to learn, live, and work. Therefore, it is vital that we improve the treatment of cognitive function in patients with depression.

At present, antidepressant drugs have no known direct effect on cognitive function. Vortioxetine is a new type of antidepressant drug that (9), in 2013, was approved for marketing by the US FDA, and in November 2017, it was approved for marketing in China by the State Drug Administration for the treatment of adult depression. Clinical studies have shown that vortioxetine can simultaneously improve the symptoms and cognitive function of patients with depression and promote the recovery of their ability to carry out social roles (10, 11). The drug can increase the levels of norepinephrine (NE) and acetylcholine by antagonizing the 5-HT3 receptor, but this only leads to an indirect improvement in cognitive symptoms through the modification of depressive symptoms (12). However, the mechanism through which vortioxetine regulates the neural circuits of cognitive networks in the brain is unclear.

A large number of multimodal magnetic resonance studies have shown that the structures, functions, and neural circuits related to cognitive function are impaired in the brains of patients with depression (13, 14). For example, structural magnetic resonance studies have found that the integrity of white matter fibers in brain regions, such as the insula, temporal lobe, and posterior cingulate gyrus, is damaged and cortical thickness is thinned in patients with depression (15–17). In recent years, fMRI technology has also been rapidly applied in clinical research into depression (18–21). Resting-state functional magnetic resonance imaging is no less important than structural magnetic resonance imaging in diagnosing brain function diseases (22). A resting-state fMRI study found abnormal amplitude of low-frequency fluctuations (ALFF) signals in the left orbitofrontal-insula circuit in patients with MDD, and abnormal ALFF values were correlated with cognitive scores in patients with depression (23). Some studies have also found that abnormal ALFF values are related to the expression of the NET-Rs28386840 gene (24, 25). The findings of these imaging studies have also revealed many new perspectives on the neuroimaging mechanisms of depression.

In terms of depression research, the following issues remain unresolved. First, studies have used different designs and focused on various aspects of brain function and therefore produced different results. For this reason, a standard format is urgently needed. Resting-state fMRI has the potential to be a standard technique for fMRI studies on clinical populations, as it is relatively easy to perform and to prevent performance-confounding factors in clinical studies. Second, the brain function of patients with MDD is often affected by antidepressants and other treatments (26–28). Therefore, in this study, we selected first-episode untreated depression patients as research subjects to eliminate these confounding factors to a large extent.

The ALFF in the BOLD signal on rs-fMRI can characterize spontaneous brain functional activity and, therefore, is often used to evaluate brain diseases such as schizophrenia and depression (29–31). In recent years, the ALFF value has proven to be a potential predictive value for evaluating the treatment outcomes in MDD patients (31). We examined ALFF alterations at two-time points (baseline and week 8 of treatment) in first-episode drug-free MDD patients at rest. Based on previous studies that reported the modulating effect of vortioxetine on cognitive function and local brain signal (32), we hypothesized that vortioxetine modulates ALFF in MDD patients. Given that previous studies reported significant correlations between alterations in brain function and symptomatic improvement, correlations between changes in ALFF values, and reductions in all the symptom scores, as rated *via* the Perceived Deficits Questionnaire for Depression (PQD-D), were expected.

## Materials and Methods

### Clinical Data

The treatment group comprised 25 patients with first-time onset and untreated MDD registered at the Wuhan Mental Health Center outpatient clinic from January 2019 to October 2020. MDD was diagnosed by a physician using the Diagnostic and Statistical Manual of Mental Disorders-Fourth Edition (DSM-IV) criteria (33). The inclusion criteria of MDD were (1) patients diagnosed with MDD for the first time less than 1 week prior to enrollment; (2) those aged between 20 and 60; (3) those with no contraindications to oral vortioxetine hydrobromide; and (4) those with no history of depressive episodes, family history of other mental illnesses, no significant physical diseases, no history of alcohol or drug abuse, and right handedness. Exclusion criteria for patients with depression and healthy controls were as follows: those with (1) organic brain disease; (2) physical severe disease; (3) family history of mental or neurological diseases; and (4) contraindications to magnetic resonance; and (5) other mental diseases or substance addictions. Twenty-five healthy controls matched by age, gender, and years of education who volunteered to participate in this study during the same period were selected. The medical ethics committee of the hospital approved this study, and all patients or their families signed informed consent.

### Scale Assessment

The 17-item version of the Hamilton Depression Scale (HAMD) and the Perceived Deficits Questionnaire for Depression (PQD-D) were used to assess the severity of illness and cognitive impairment in all patients (34).

### Treatment Methods

The treatment group was given oral vortioxetine hydrobromide tablets (trade name: Xindayue, Lundbeck, Beijing; Pharmaceutical Information Consulting Co., Ltd., approval number: H20170382). The initial and maintenance doses were 10–20 mg/d. During the study period, other antidepressants, antipsychotics, sedative-hypnotics, and other psychiatric drugs were prohibited.

### Data Collection

Rs-fMRI scans were performed with a Philips 3.0 MRI machine. The subjects were instructed to relax, breathe calmly, close their eyes, and keep their minds awake during the scan. All subjects underwent rs-fMRI scans with a scanning sequence as follows: (1) structural image adoption of 3D spoiler gradient echo, TR = 8.4 ms, TE = 3.2 ms, flip angle = 12°, slice thickness = 1 mm, FOV = 24 cm × 24 cm, matrix = 256 × 256, and a scanning time of about 15 min; (2) rs-fMRI imaging parameters were echo plane imaging sequence, TR = 2,000 ms, TE = 40 ms, FOV = 24 cm × 24 cm, matrix = 60 × 60, flip angle = 90°, slice thickness = 4 mm, layer spacing = 0 mm, number of layers = 33, and a scanning time of about 11 min.

### Data Processing

The data were processed using the DPABI2.2 and SPM12 toolkits on the Matlab platform (35). The processing steps were as follows: first, DICOM map was converted to NIFTI format; the 10 time points were removed at the beginning of the scan to obtain the stability of the image acquisition; then the functional image was resampled and registered to the structural image of each subject. This was followed by Gaussian smoothing of 6 mm × 6 mm × 6 mm. Full-width and half-height de-linear drift and filtering were performed to extract signals in the frequency range of 0.01–0.08 Hz. Finally, the ALFF value of each voxel in the whole brain was calculated and divided by the average ALFF value of the entire brain to obtain the standard ALFF value.

### Statistical Analysis

SPSS22.0 was used for statistical analysis of general demographic data and symptom scores. Statistical analysis software with REST was employed to perform statistical analysis on ALFF images. Firstly, an analysis of the results for the three groups of subjects was carried out. Then, two independent-sample *t*-tests were carried out on the healthy group results before and after treatment. The Rest Slice Viewer was used for multiple comparison corrections and the obtained images. Differences in brain regions with *P* < 0.01 were defined as statistically significant. The correlation between significant changes in the ALFF value and HAMD score or disease course were analyzed.

## Results

There were no statistically significant differences in gender, age, or years of education between the depression patient group (25 patients before and after treatment) and the standard control group. There were statistically significant decreased scores in HAMD and PQD-D after treatment (Table 1).

**TABLE 1:** Demographic information.

Characteristics	Pre-treatment patient (*n* = 25)	Post-treatment patient (*n* = 25)	Controls (*n* = 25)	*F* or ×2 or *T*	*P*-value
Gender (male/female)	25 (6/19)	25 (6/19)	25 (7/18)	18.25	>0.05
Age (years)	28.80 ± 7.54	28.80 ± 7.54	27.24 ± 8.17	0.34	>0.05
Education (years)	11.24 ± 2.58	11.24 ± 2.58	12.80 ± 2.46	3.13	>0.05
HAMD scores	26.40 ± 5.29	11.20 ± 1.25		4.21	0.03
PQD-D scores	52.15 ± 11.24	38.85 ± 11.14		4.35	0.04

*HAMD, Hamilton Rating Scale for Depression; PQD-D, Perceived Deficits Questionnaire for Depression.*

**FIGURE 1:**
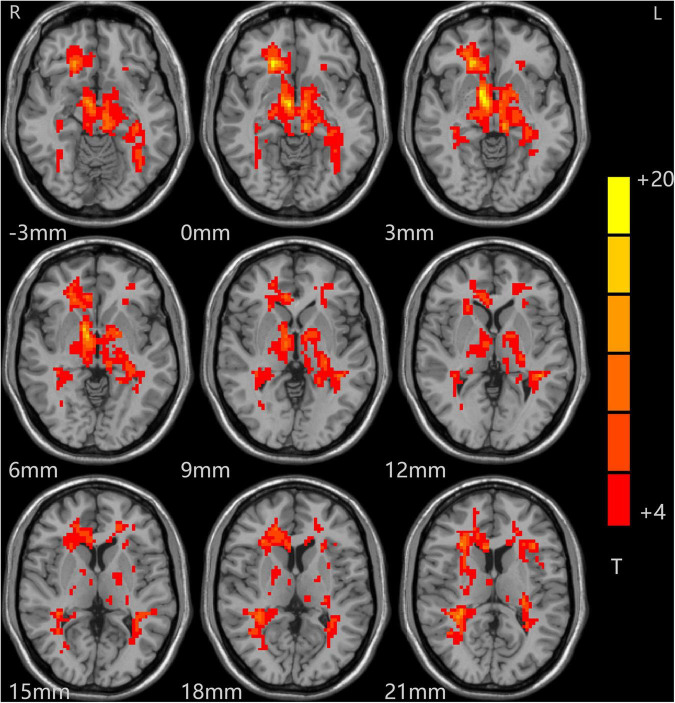
Alterations in ALFF among patients (at baseline, after treatment) and controls. Red indicates the brain area where ALFF values were significantly increased, and the color depth indicates the differences between the three groups.

The analysis of variance between the ALFF values of the depression group and the control group before and after treatment showed that the ALFF values of the bilateral middle frontal gyrus (MFG), cingulate gyrus (CG), and middle temporal gyrus (MTG) increased significantly in the depression group, as shown in Table 2 and Figure 1.

**TABLE 2:** Alterations of ALFF among patients (at baseline, after treatment) and controls.

Cluster location	Peak (MNI)			Number of voxels	*T*-value
	*X*	*Y*	*Z*		
Bilateral MFG	36	−15	−51	33	7.64
Bilateral CG	9	−6	−6	28	2.18
Bilateral MTG	−30	12	18	−27	8.62

*ALFF, amplitude of low-frequency fluctuation MFG, middle frontal gyrus; CG, cingulate gyrus; MTG, middle temporal gyrus.*

**FIGURE 2:**
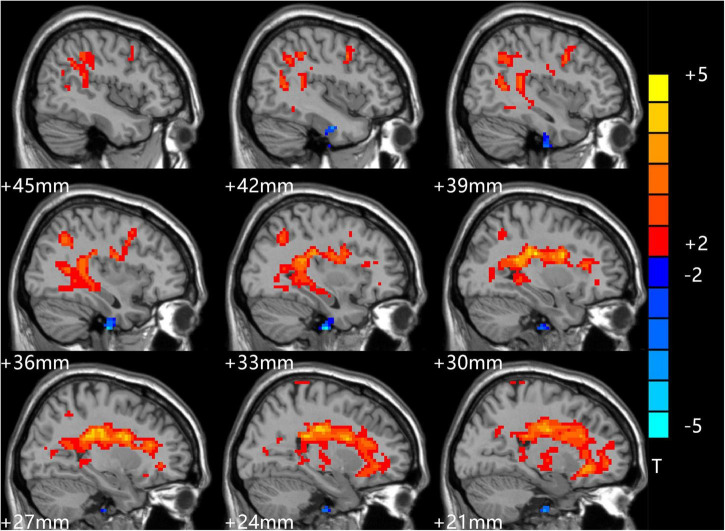
Comparison of patients and controls at baseline. Blue indicates the brain area where ALFF values were significantly reduced. Red indicates the brain area where ALFF values were significantly increased, and the color depth indicates the difference between the two groups.

The *t*-test results comparing two independent samples from the depression group before treatment and the standard control group showed that ALFF values in the right inferior temporal gyrus (ITG) decreased significantly, while those in the left low cerebellum, right CG, and central posterior gyrus (CPG) increased dramatically and significantly in the patient group, as shown in Table 3 and Figure 2.

**TABLE 3:** Alterations of ALFF between patients at baseline and controls.

Cluster location	Peak (MNI)			Number of voxels	*T*-value
	*X*	*Y*	*Z*		
Right ITG	33	−15	−51	67	−3.60
Left cerebellum	−27	−30	−33	28	3.868
Right CG	12	3	−3	49	6.23
Right CPG	15	−51	78	32	4.11

*ALFF, amplitude of low-frequency fluctuation; ITG, inferior temporal gyrus; CG, cingulate gyrus; CPG, central posterior gyrus.*

**FIGURE 3:**
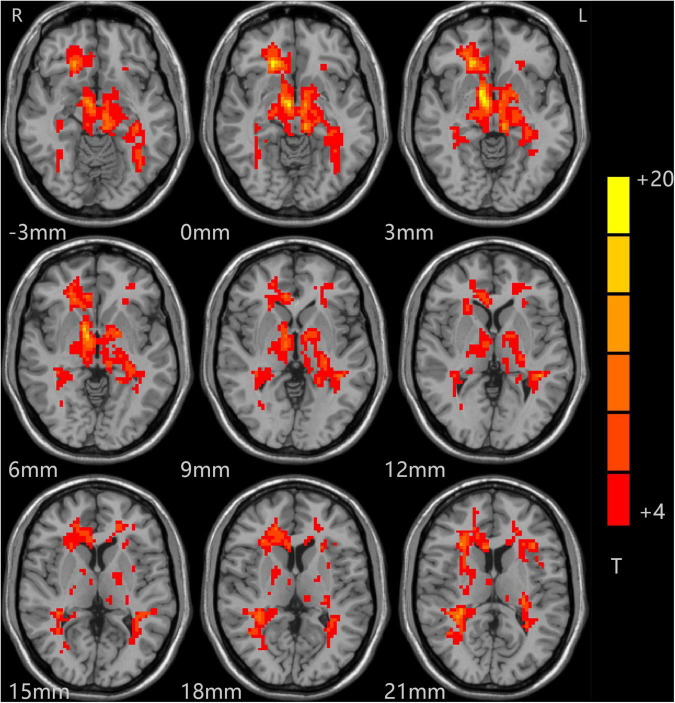
Comparison of patients and controls after treatment. Red indicates the brain area where ALFF values were significantly increased, and the color depth indicates the difference between the two groups.

After treatment, the *t*-test results of two independent samples from the depression group and control group showed that ALFF values increased in the bilateral CG, bilateral MTG, bilateral medial superior frontal gyrus (MSFG), and bilateral inferior cerebellum in patients in the depression group, as shown in Table 4 and Figure 3.

**TABLE 4:** Alterations of ALFF between patients after treatment and controls.

Cluster location	Peak (MNI)			Number of voxels	*T*-value
	*X*	*Y*	*Z*		
Bilateral CG	18	30	51	36	3.29
Bilateral MTG	21	30	−27	30	3.50
Bilateral MSFG	6	63	33	63	3.45
Bilateral cerebellum	39	−30	−9	65	6.56

*ALFF, amplitude of low-frequency fluctuation; CG, cingulate gyrus; MTG, middle temporal gyrus; MSFG, medial superior frontal gyrus.*

## Discussion

In this study, the ALFF analysis method proposed by Yan et al. (36) was used to study brain functional changes in patients with depression before and after treatment with vortioxetine. The brain regions with increased ALFF values included the left inferior cerebellum, right cingulate gyrus (CG), and posterior central gyrus (PCG). In this study, data from a study of first-episode patients with depression without antidepressant drugs and various treatments were collected (37–39). After 8 weeks of standard vortioxetine antidepressant therapy, brain areas with significantly decreased spontaneous activity in patients with depression were corrected. The brain areas with considerably increased ALFF values were mainly distributed in the bilateral CG, medial superior frontal gyrus (MSFG), middle temporal gyrus (MTG), cerebellum, and right inferior temporal gyrus (ITG), middle frontal gyrus (MFG). Our findings showed that vortioxetine can effectively control depressive symptoms and improve cognitive function in patients with MDD.

In this study, the areas of brain functional changes in patients with depression were mainly concentrated in the frontal lobe, temporal lobe, CG, and ITG. A large number of studies have shown that the frontal lobe has key roles in attention, emotional processing, reward and cognitive punishment functions, and is involved in the inhibition of various behaviors (40). A near-infrared imaging study found changes in functional signals in the MFG in patients with major depression, and a momentary mood found correlations between rumination responses during induced stress and everyday stress rumination (41). Depressed mood and delayed thinking in patients with depression may be related to the development of frontal lobe dysfunction, resulting in poor prognosis and co-morbid psychiatric disorders. The temporal lobe and CG are important components of the default and limbic networks (42), which are involved in the human brain’s emotional processing and self-control functions, and they indirectly connect important areas affecting the frontal lobe through the Papez circuit, mainly the corticostriatal-pallidus-thalamic neuroanatomical circuit (43). A multicenter study found differences in resting functional connectivity in the emotional circuit of the marginal structure frontal lobe striatum thalamus between unipolar and bipolar depressive disorder brains (44). Another meta-analysis found distinct patterns of intrinsic brain activity alterations in the local brains of patients with monophasic and bipolar depression (45). Therefore, there are certain abnormalities in the brain function networks of patients with depression, mainly concentrated in the temporal lobe and CG. In addition to functional changes, there are also corresponding structural changes in these brain regions. Structural magnetic resonance imaging showed that patients with depression had stronger deep white matter signals and decreased gray matter volume in the cortex and anterior cerebellar lobe (46). These studies suggested that marginal networks are directly involved in the pathogenesis of depression.

The CG has been an important role subject of focus in previous pathological studies of depression, and the signs seen during the pathogenesis of depression are different between the anterior CG and posterior CG. The anterior CG is mainly related to self-control and emotional control, while the posterior CG is primarily related to early awakening, introspection, memory, and thought flexibility (47). Differences in the signs of depression between the two may be related to the core brain regions of the posterior CG involved in the default network. The default network forms connections between brain areas that are active when the patient is in a quiescent state and there are no tasks or other forms of stimulation. It is believed to be related to human emotional regulation as well as attention and sleep circadian rhythm disorders. There are various research reports on the role of the CG in depression, but there are also opposing views. For example, Fan and his colleagues found decreased VMHC in the bilateral posterior cingulate cortex (PCC) extending to the precuneus in patients with MDD compared with healthy controls (48). Relative to healthy controls, melancholic patients also displayed decreased VMHC in the PCC (49). However, a recent meta-analysis MDD of the PCC showed higher FC in the bilateral MTG (47). The inconsistencies in these research results may have been caused by the different sample sizes, analysis methods, and software used, and the lack of a guarantee of complete homogeneity in the subjects selected. For example, the course of pathogenesis in some patients is inconsistent with what is typical with depression, and some signs are caused by interference from drug treatment, psychotherapy, or physical therapy (50–54). The right ITG in the human brain is related to depression and decreased pleasure, but it is also directly involved in memory, appetite, and other functions, as well as being indirectly involved in cognitive functions such as attention and vigilance.

Based on the results of this study, we inferred that the decrease in ALFF values in the right ITG may be related to depression. The new antidepressant vortioxetine has multiple modes of action that can comprehensively improve the depressive symptoms of patients. Clinical observations provide support for the drug’s significant curative effects, including its ability to reduce the frequency of depression recurrence and improve patients’ social and cognitive function (32). Our study found that the ALFF values of the right ITG of patients returned to normal after vortioxetine antidepressant treatment, which may be related to the concurrent improvements in depressive symptoms. This provides a biological mechanism for the effect of vortioxetine as an antidepressant drug that can effectively improve cognitive function from the perspective of neuroimaging.

Over recent years, scholars have found that the cerebellum plays a specific role in the default network. Guo Wenbin’s team found enhanced signals of cerebellar functional connection in patients with depression, and this compensatory mechanism is used to resist the early clinical symptoms of patients with depression (55). Our study also found spontaneous signal enhancement in the cerebellum after treatment. Therefore, we speculated that the changes in cerebellar spontaneous activity in patients with depression may be related to their symptoms of negative thinking and depression.

There were some limitations to this study. First, the sample size was too small for satisfactory statistical power. Second, we did not exclude patients with bipolar disorder, who may only show depression in the early stages of the condition. These factors may have affected the stability of the research results. Lastly, this report only discusses differences in spontaneous signals occurring in the whole brain of patients with depression from the perspective of low-frequency amplitude functional magnetic resonance and speculates on the pathophysiological mechanisms of depression and the possible antidepressant and cognitive function mechanisms of votioxetine. In the future, it will be beneficial to further explore the specific diagnosis and treatment of depression from multiple perspectives.

## Conclusion

This study used resting magnetic resonance imaging technology and the ALFF analysis method to explore brain functional changes in first-episode drug-free patients with MDD before and after vortioxetine treatment to provide an objective basis for the diagnosis and treatment of patients with MDD.

## Data Availability Statement

The original contributions presented in this study are included in the article/supplementary material, further inquiries can be directed to the corresponding author/s.

## Ethics Statement

The studies involving human participants were reviewed and approved by the Medical Ethics Committee of the Hospital approved this study, and all patients or their families signed informed consent. The patients/participants provided their written informed consent to participate in this study.

## Author Contributions

All authors listed have made a substantial, direct, and intellectual contribution to the work, and approved it for publication.

## Conflict of Interest

The authors declare that the research was conducted in the absence of any commercial or financial relationships that could be construed as a potential conflict of interest. The reviewer XJ declared a shared affiliation with the author SZ at the time of review.

## Publisher’s Note

All claims expressed in this article are solely those of the authors and do not necessarily represent those of their affiliated organizations, or those of the publisher, the editors and the reviewers. Any product that may be evaluated in this article, or claim that may be made by its manufacturer, is not guaranteed or endorsed by the publisher.
